# The survival analysis and oncogenic effects of CFP1 and 14-3-3 expression on gastric cancer

**DOI:** 10.1186/s12935-019-0946-3

**Published:** 2019-08-31

**Authors:** Jingyue Sun, Yao Long, Xin Peng, Desheng Xiao, Jianhua Zhou, Yongguang Tao, Shuang Liu

**Affiliations:** 1 Key Laboratory of Carcinogenesis and Cancer Invasion, Ministry of Education, Department of Pathology, Xiangya Hospital, Central South University, Hunan, 410078 China; 2 NHC Key Laboratory of Carcinogenesis of Ministry of Health (Central South University), Cancer Research Institute, School of Basic Medicine, Central South University, Changsha, 410078 Hunan China; 3 Department of Pathology, Xiangya Hospital, Central South University, Changsha, 410008 Hunan China; 4 Hunan Key Laboratory of Tumor Models and Individualized Medicine, Department of Thoracic Surgery, Second Xiangya Hospital, Central South University, Changsha, China; 5 Department of Oncology, Institute of Medical Sciences, Xiangya Hospital, Central South University, Changsha, 410008 Hunan China

**Keywords:** Gastric cancer, CFP1, 14-3-3

## Abstract

**Background & aim:**

Gastric cancer (GC) is the third-leading cause of cancer-related deaths. We established a prospective database of patients with GC who underwent surgical treatment. In this study, we explored the prognostic significance of the expression of CFP1 and 14-3-3 in gastric cancer, by studying the specimens collected from clinical subjects.

**Materials & methods:**

Immunohistochemistry was used to detect the expression of CFP1 and 14-3-3 in 84 GC subjects, including 73 patients who have undergone radical gastrectomy and 11 patients who have not undergone radical surgery. Survival analysis was performed by km-plot data.

**Results:**

According to the survival analysis, we can see that the survival time of patients with high expression of CFP1 is lower than the patients with low expression in gastric cancer, while the effect of 14-3-3 is just the opposite. The survival time of patients with higher expression of 14-3-3 is also longer.

**Conclusion:**

The CFP1 and 14-3-3 genes can be used as prognostic markers in patients with GC, but the study is still needed to confirm.

## Background

Gastric cancer (GC) is one of the most frequently occurring malignancies worldwide and the third-leading cause of cancer-related deaths worldwide [1]. The 5-year survival rate of gastric cancer is less than 30% [2–4]. Tumor metastasis is the most important cause of death. Surgery is the main treatment, and the median survival time varies with different postoperative chemotherapy combinations [5–7]. Many studies have studied molecular markers of gastric cancer, and the mechanism of gastric cancer has been well understood, but its prognosis is still poor. So we urgently need to detect new markers and therapeutic targets for gastric cancer [8–17].

The CXXC zinc finger protein 1 (CFP1, also known as CGBP) is a subunit of the TrxG SET1 protein complex, a major catalyst of histone 3 lysine 4 trimethylation (H3K4me3) [18, 19]. CFP1 binds to DNA via its CXXC finger domain and its PHD domain, and recruits SETD1 to the promoter of actively transcribed CGI-related genes [20]. It has been reported that some cells lacking CFP1 may not mature and fail to function, such as oocytes [21, 22]. CFP1 is a specific factor that integrates multiple signals, including promoter CpG content and gene activity, to regulate the genome-wide pattern of H3K4me3 [23–25]. Therefore, the loss of CFP1 may have effects on the function and maturation of cells, and may promote the development of tumors.

The 14-3-3 family proteins comprise seven isoforms. They exist as dimers (homo- or hetero-dimer) in cells [26]. 14-3-3 proteins interact with a broad spectrum of proteins involved in cell signaling, transcriptional regulation, cytoskeletal remodeling, DNA repair and apoptosis. Thus, 14-3-3 proteins regulate a variety of cellular functions, including cell cycle, cell development, cell proliferation, and cell movement [27]. 14-3-3 proteins can regulate the structure of their targets and other factors, stability, intracellular localization and interaction,and its mutation is associated with many human cancers [26–30].

Although studies about gastric cancer have found some markers, such as HER2, CEA and many microRNAs, gastric cancer is still a tumor with high mortality, and its incidence is high. From the literature, it can be found that both CFP1 and 14-3-3 have effects on the function of cells, and there is a relationship with development of some tumors. The two genes have not been linked to gastric cancer in the existing literature. So we studied the effects of CFP1 and 14-3-3 on the survival time of gastric cancer through clinical samples of 84 cases, KM-plot and TCGA database.

## Materials & methods

### Patients in the study

Our research group established a prospective database for gastric cancer since 2015, and information in 84 cases of gastric cancer has been collected. Between January 2015 and December 2015, all subjects with gastric cancer were treated by surgeon at the Xiangya Hospital. The data used in this experiment was used in the case of honoring patient-physician confidentiality, which protected the patient’s privacy and met the ethical requirements and was approved by the Ethics Committee of the Cancer Institute of Central South University. About 73 subjects of these were treated by Radical gastrectomy, the others are treated by Exploratory laparotomy. About the 84 gastric cancer subjects included 53 males and 31 females, aged 31–75 years (a median age of 58 years), with stage I (n = 21), II (n = 23), III (n = 20), and IV (n = 20) diseases according to the criteria of the Tumor & regional lymph node & metastasis (TNM) classification system of malignant tumors. In addition, we obtained 373 cases of gastric cancer through the TCGA database, including 30 normal tissues and 343 gastric cancer tissues, and prepared a heat map.

### Immunohistochemical staining

After routine deparaffinization and hydration, tissue sections were treated with 3% hydrogen peroxide and then heated in sodium citrate for antigen retrieval. After antigen retrieval, the activity of endogenous peroxidase was eliminated by 3%H_2_O_2_. Then, antibodies used were as follows: anti-CFP1 (1:500); anti- 14-3-3-IHC (1:500), 4 °C overnight, followed by incubation with the corresponding secondary antibody at room temperature for 30 min. All stained slides were initially reviewed and scored by the first author and re-viewed by three pathologists in a blinded fashion to ensure consistency of interpretation. To assess CFP1 and 14-3-3 expression, immunohistochemical staining was divided into the following four groups according to intensity and degree. The slides were first scored as 0 (negative), 1 (buff), 2 (pale brown), and 3 (tan). Positive expression of CFP1 and 14-3-3 were scored as 0 (negative), 1 + (< 10% of positively-staining tumor cells), 2 + (11–50% of positively-staining tumor cells), 3 + (50–75% of positively-staining tumor cells), and 4 + (> 75% of positively-staining tumor cells. Both the scores by multiply were regarded as the determination result.

### Follow-up information

We then followed up on the 84 cases. Unfortunately, nearly half of the patients lost to follow-up. Of the 33 follow-up cases, 27 received radical surgery and the remaining 6 patients underwent laparotomy. Fifteen of the 33 patients received postoperative chemotherapy, while the remaining 18 did not receive chemotherapy. So far, 15 patients have died, and the remaining 18 have been in good health according to the follow-up results.

### Statistical methods

Statistical analysis was performed using SPSS 17.0 statistical software. The TNM classification system of malignant tumors was used in our study. The expression of CFP1 and 14-3-3 and clinicopathologic characteristics was tested by X^2^ test. The survival analysis was carried out by KM plotter. P values < 0.05 were considered statistically significant.

## Results

**Fig. 1:**
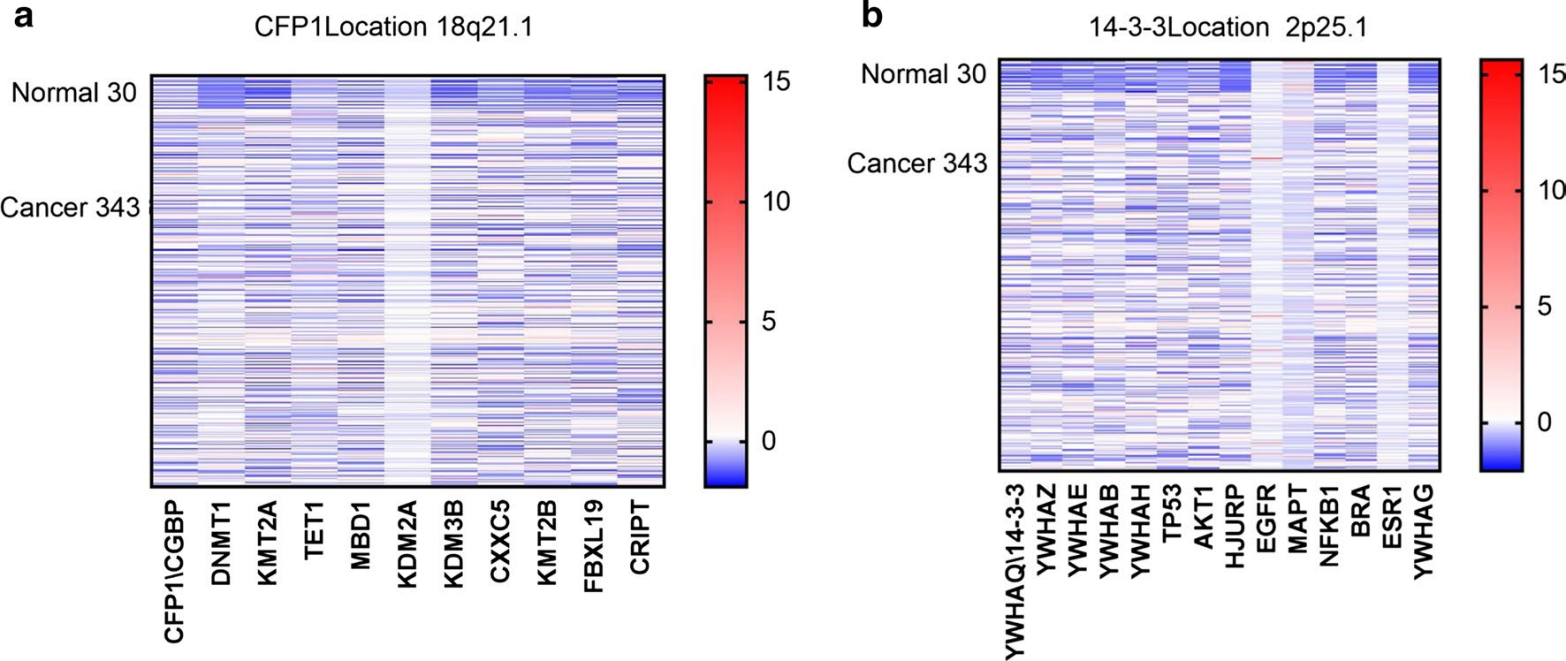
Heat map. The vertical axis of the heat map indicates the number of cases, the first 30 is normal gastric tissue, and the last 343 is gastric cancer tissue. We used the data from the TCGA database to draw heat maps for the same family genes and related genes for CFP1 or 14-3-3. **a** It can be seen that in the heat map drawn by CFP1 and other genes of its same family, CFP1 expression in normal tissues is lower than in tumor tissues. **b** It can be seen that in the heat map of 14-3-3 homologous genes and related genes, the expression of 14-3-3 in normal tissues is lower than that in tumor tissues

**Fig. 2:**
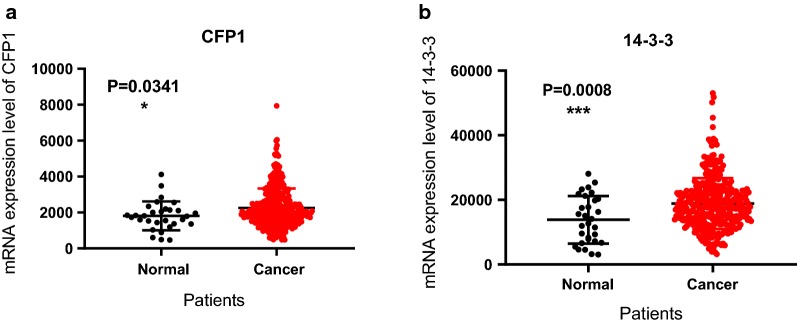
The mRNA expression of CFP1 and 14-3-3 in 30 normal gastric tissues and 343 gastric cancer tissues. **a** Using data from the TCGA database to analyze the expression of CFP1 in normal and gastric cancer tissues, we can see that CFP1 is expressed more highly in gastric cancer, and the results are statistically significant (P < 0.05). **b** Using data from the TCGA database to analyze the expression of 14-3-3 in normal and gastric cancer tissues, we can find that 14-3-3 is expressed more highly in gastric cancer, and the results are statistically significant (P < 0.05)

In order to find new markers and therapeutic targets for gastric cancer, we obtained some genes expressed in gastric cancer through the TCGA database, and selected a part of the same family and related genes to draw heat maps (Fig. 1). The data obtained from the TCGA database included 30 normal tissues and 343 gastric cancer tissues. It can be seen from the heat map that there is a certain change in the expression of CFP1 and 14-3-3 in tumor tissues compared with the same family genes and related genes. We mapped the expression differences of CFP1 and 14-3-3 between normal and gastric cancer patients by the data we obtained from TCGA (Fig. 2). It can be seen that the expression of CFP1 and 14-3-3 in gastric cancer tissues is higher than that in normal gastric tissues, and this difference was statistically significant (P < 0.05).

**Fig. 3:**
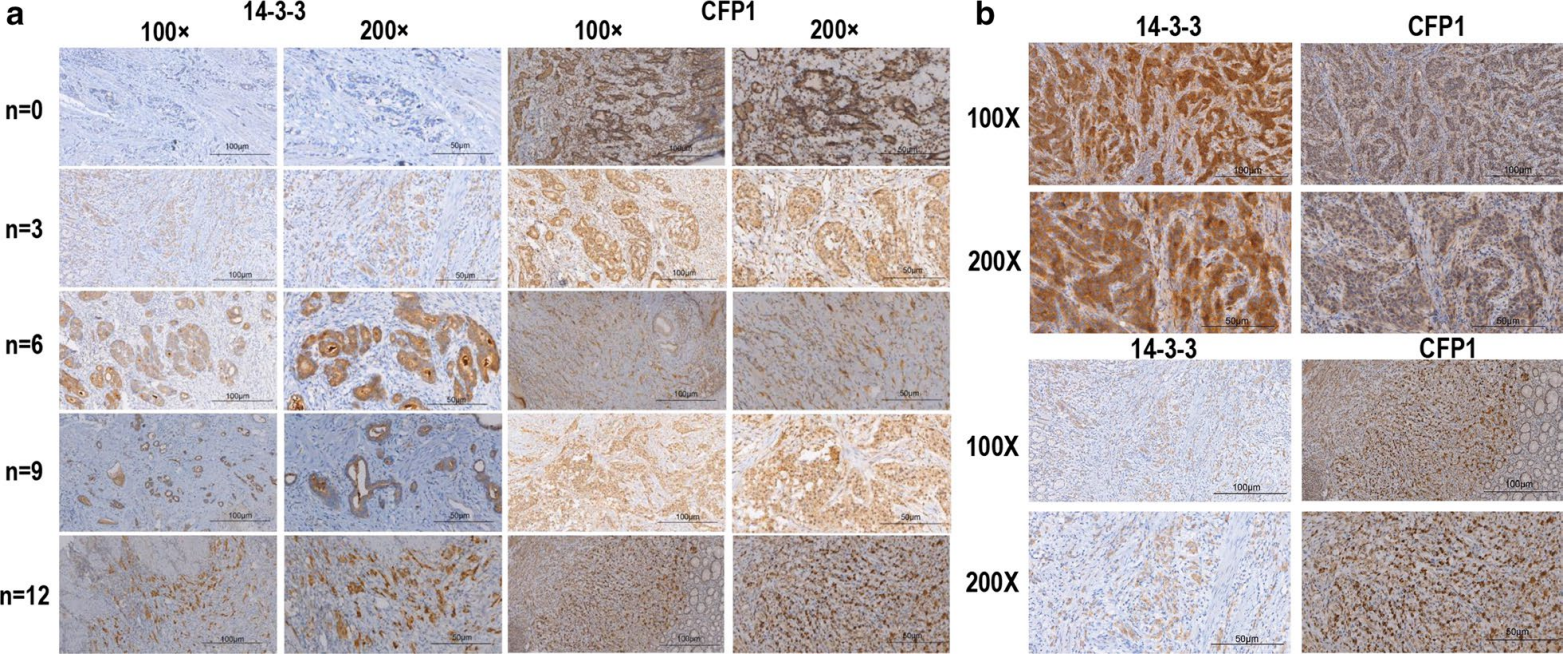
The immunohistochemistry of clinical gastric cancer tissue. Immunohistochemistry was performed on clinically obtained gastric cancer samples. **a** The immunohistochemistry images of 14-3-3 and CFP1 expression seen under microscopes at 100X and 200X, and the expression of CFP1 and 14-3-3 in gastric cancer was scored (n = 0, 3, 6, 9, 12) **b** Up: The expression of 14-3-3 was higher in the same field of view, while CFP1 was lower. Down: the expression of CFP1 was higher in the same field of view, and the expression of 14-3-3 was low

To confirm this, we selected 84 clinical samples for immunohistochemistry. By immunohistochemistry of clinical tissue, we found that CFP1 is mainly expressed in the nucleus, while 14-3-3 is mainly expressed in the cytoplasm. We scored the clinical tissues according to the level of immunohistochemical expression at 0, 3, 6, 9, and 12. By immunohistochemistry, it can be seen that when the expression of 14-3-3 is high, the expression of CFP1 in the same visual field is low, and when the expression of CFP1 is high, the expression of 14-3-3 is low (Table 1, Fig. 3). If we assume that the score is higher than six, it is high. Among the 84 subjects in the study, 53.6% (45/84), 64.3% (54/84) had high-level expression of CFP1 and 14-3-3.

**Table 1:** Association between CFP1 and 14-3-3 expression and clinical characteristics of 84 patients with GC

	CFP1 expression No.					P	14-3-3 expression No.					P
	0	3	6	9	12		0	3	6	9	12	
Age												
≥ 58	2	21	11	4	6	0.196	0	14	21	4	5	0.012
< 58	4	12	7	9	8		2	14	7	12	5	
Sex												
Male	4	23	12	6	8	0.633	1	15	20	9	8	0.46
Female	2	10	6	7	6		1	13	8	7	2	
Tumor invasion (T)												
T1	4	11	2	0	0	0	0	7	7	3	0	0.576
T2	1	3	3	0	0		0	1	4	0	2	
T3	1	13	7	4	2		1	7	9	6	4	
T4	0	6	6	9	12		1	13	8	7	4	
Lymphnode metastasis (N)												
N0	4	21	9	2	1	0.001	0	12	18	6	1	0.343
N1	1	4	2	3	1		1	4	2	2	2	
N2	1	1	1	2	0		0	2	2	0	1	
N3	0	4	5	2	3		1	5	3	3	2	
Nx	0	3	1	4	9		0	5	3	5	4	
Metastasis (M)												
M0	6	30	16	7	5	0	2	22	24	10	6	0.266
M1	0	3	2	6	9		0	6	4	6	4	
Stage												
I	4	13	4	0	0	0	0	7	10	3	1	0.242
II	2	11	6	2	2		0	7	10	4	2	
III	0	6	6	5	3		2	8	4	3	3	
IV	0	3	2	6	9		0	6	4	6	4	
R0 resection												
Yes	6	31	17	10	9	0.031	2	25	25	14	7	0.542
No	0	2	1	3	5		0	3	3	2	3	
Postoperative chemotherapy												
Yes	0	13	10	7	6	0.155	2	9	12	7	6	0.314
No	2	10	0	2	3		0	5	8	4	0	
Uncertain	4	10	8	4	5		0	14	8	5	4	
Level of differentiation												
Well	0	3	0	1	0	0.518	0	3	1	0	0	0.761
Middle	2	11	4	5	2		1	8	9	4	2	
Poor	4	19	14	7	12		1	17	18	12	8

**Fig. 4:**
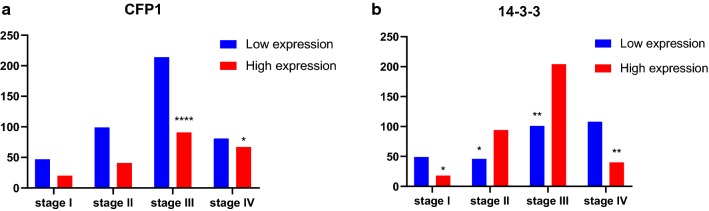
The differential analysis of the expression levels of CFP1 and 14-3-3 in stage I–IV by data obtained by KM-plot. Combined with the data and graphs in Table 3, it can be seen that with the upgrade of gastric cancer, the proportion of those with high CFP1 expression is gradually increasing. In addition, it can be found that the expression level of CFP1 and 14-3-3 shows an opposite trend in the III and IV phases. *Represents the correlation between high and low expression of genes in the same stage

Based on the results obtained above, we used Kaplan–Meier analysis the CFP1 and 14-3-3 association with survival time. It can be seen that the median survival time of the high expression of CFP1 was 9.33 months and the median survival time of low expression of CFP1 was 22 months. The median survival time of patients with high expression of 14-3-3 was 85.8 months, and the median survival time of low expression was 25.2 months (Table 2). Survival analysis by Kaplan–Meier data shows that in the I–IV cases, the high expression of 14-3-3 and CFP1 have different effects on the median survival time of the cases (Table 3, Fig. 4).

**Table 2:** CFP1 and 14-3-3 expression in 876 GC tissues and association with overall survival time

Item	No. (%)	Median survival time, mo (95% confidence interval)	P
CFP1			
Low expression	621 (70.9)	–	< 0.0001
High expression	255 (29.1)	23.43	
14-3-3			
Low expression	233 (26.6)	25.2	< 0.0001
High expression	643 (73.4)	85.8

**Table 3:** TNM stage-stratified analysis between CFP1 and 14-3-3 expression and overall survival time in 660 GC tissues

Item	No. (%)	Median survival time, mo (95% confidence interval)	P
Stage I			
CFP1 low expression	47 (70.1)	–	0.0301
CFP1 high expression	20 (29.9)	–	
14-3-3 low expression	49 (73.1)	93.2	0.0190
14-3-3 high expression	18 (26.9)	–	
Stage II			
CFP1 low expression	99 (70.7)	–	0.0317
CFP1 high expression	41 (29.3)	123.6	
14-3-3 low expression	46 (32.9)	123.6	0.0258
14-3-3 high expression	94 (67.1)	123.8	
Stage III			
CFP1 low expression	214 (70.2)	89.43	< 0.0001
CFP1 high expression	91 (29.8)	24.5	
14-3-3 low expression	101 (33.1)	25.9	0.0015
14-3-3 high expression	204 (66.9)	47.7	
Stage IV			
CFP1 low expression	81 (54.7)	20.03	0.0386
CFP1 high expression	67 (45.3)	14.3	
14-3-3 low expression	108 (73.0)	11.4	0.0032
14-3-3 high expression	40 (27.0)	20.03

**Fig. 5:**
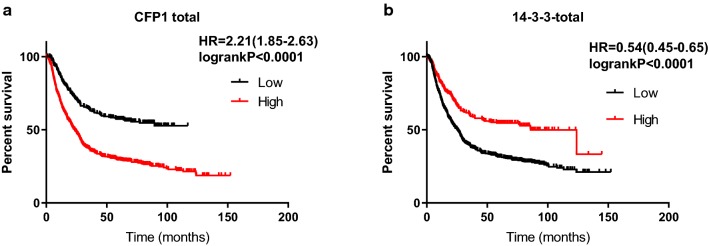
Survival curve produced by data obtained by KM-plot. **a** The survival time of patients with high expression of CFP1 is lower than that of patients with low expression. (P < 0.0001). **b** Among the patients with high expression of 14-3-3, the survival time is lower than patients with high expression (P < 0.0001)

**Fig. 6:**
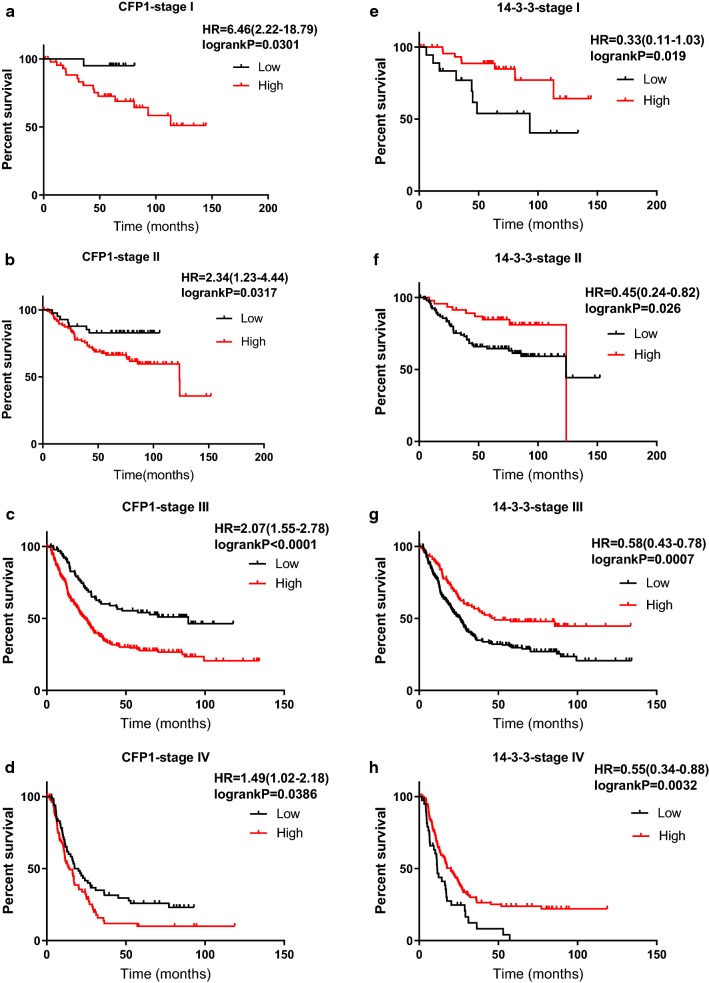
Survival analysis of gastric cancer specimens of stage I–IV gastric cancer by KM-plot data. **a**–**d** CFP1 has a negative effect on survival time in stage I-IV. The survival time of high expression is lower than that of low expression (P < 0.05). **e**–**h** 14-3-3 has a positive effect on survival time in stage I-IV. The survival time of high expression is longer than that of low expression (P < 0.05)

**Fig. 7:**
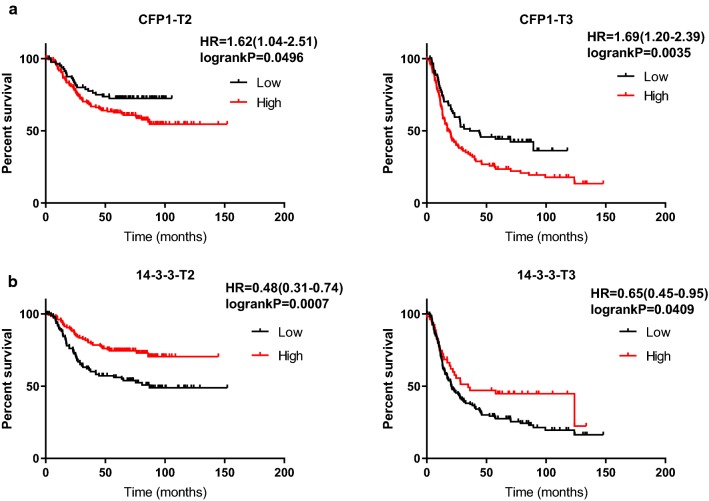
Survival analysis of gastric cancer specimens of different stages by KM-plot data. **a** In T stage, T1 does not have enough data for survival analysis, and we can see the survival time of gastric cancer patients with high expression of CFP1 is short in T2 and T3(P < 0.05). **b** The survival time of gastric cancer patients with high expression of 14-3-3 is longer in T2 and T3(P < 0.05)

**Fig. 8:**
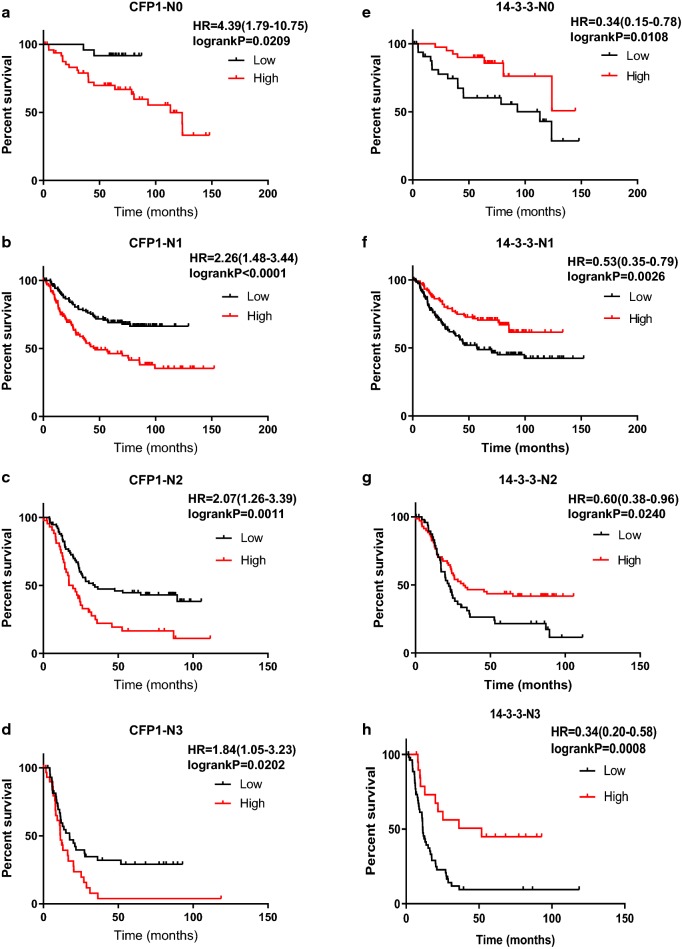
Survival analysis of gastric cancer specimens of lymph node metastasis stage by KM-plot data. **a** In lymph node metastasis stage N0–N3, the survival time of gastric cancer patients with high expression of CFP1 is shorter than the patients with low expression (P < 0.05). **b** In lymph node metastasis stage N0–N3, the survival time of gastric cancer patients with high expression of 14-3-3 is longer than the patients with low expression (P < 0.05)

**Fig. 9:**
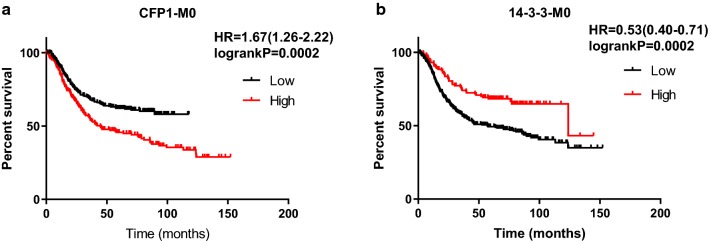
Survival analysis of gastric cancer specimens of metastasis stage by KM-plot data. **a** In patients without distant metastases, the survival time of the patients with low expression of CFP1 is longer than the patients with high expression. P value is less than 0.05. **b** In patients without distant metastases, the survival time of the patients with low expression of 14-3-3 is shorter than the patients with high expression. P value is less than 0.05

**Fig. 10:**
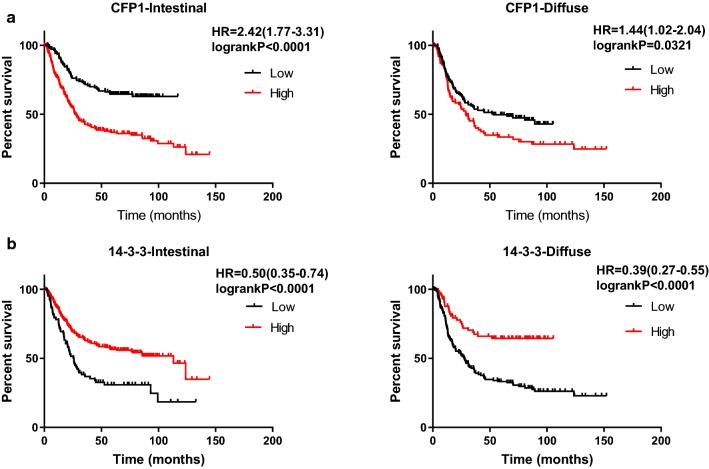
Survival analysis of gastric cancer specimens of Lauren’s classification by KM-plot data. **a** In the survival analysis of Lauren’s classification, it can be seen that patients with intestinal and diffuse gastric cancer, CFP1 still has a negative effect on survival time (P < 0.05). **b** In the survival analysis of Lauren’s classification, among the patients with intestinal and diffuse gastric cancer, 14-3-3 still has a positive effect on survival time (P < 0.05)

We used Kaplan–Meier to analyze the effect of CFP1 and 14-3-3 on survival time in gastric cancer. In general, the survival time of patients with high expression of CFP1 in gastric cancer is lower than patients with low expression of CFP1, while patients with high expression of 14-3-3 have better prognosis than patients with low expression (Fig. 5). According to the patient’s TNM staging, the patients can be divided into I–IV phases, and we used the data obtained by KM-plot to map the survival analysis. As can be seen from the figure, in the I–IV phase, high expression of CFP1 has a negative effect on prognosis, while 14-3-3 plays a positive role (Fig. 6). We only could draw the survival curve of T2-T4 phases due to insufficient data of T1 phase. Because the survival stage of the T4 phase was not statistically significant, we only used the survival analysis of the T2 and T3 phases. We can find that CFP1 is negative for survival time in T2 and T3, while 14-3-3 is the opposite (Fig. 7). In the lymph node metastasis of tumor, the effect of CFP1 and 14-3-3 is the same as describe above (Fig. 8). In cases without distant metastases, the patients with high expression of CFP1 have shorter survival time, and the patients with high expression of 14-3-3 have longer survival time (Fig. 9). However, the difference was not statistically significant in M1 cases. In Lauren’s classification, the difference in survival time between CFP1 and 14-3-3 in intestinal and diffuse cases was statistically significant, but it was not statistically significant in the mixed type (Fig. 10).

In summary, we can conclude that CFP1 and 14-3-3 have a certain impact on the prognosis in gastric cancer, which is consistent with our expectation.

## Discussion

### The main treatment for gastric cancer is still surgery

Almost all patients with gastric cancer will undergo surgery. Of the 84 cases, 73 have undergone radical surgery. The other patients who have not undergone radical surgery have had distant metastases and the lesions are diffuse. According to the patient’s condition, some patients received postoperative chemotherapy and some did not. However, preoperative neoadjuvant chemotherapy is still not common therapy. Only one of the 84 patients received preoperative chemotherapy. It is impossible to determine whether preoperative neoadjuvant chemotherapy will have a certain impact on the patient’s prognosis and gene expression. But nowadays, gastric cancer is still one of the major cancers causing human death, so we need to find new and effective targets that will have a positive impact on the patient’s prognosis and survival time.

### 14-3-3 and CFP1 also have a role in other tumors

According to the existing literature and research, we can find that CFP1 and 14-3-3 play a certain role in different tumors.14-3-3 proteins are positive regulators of the tumor suppressor p53, the mutation of which is implicated in many human cancers [30]. There are seven 14-3-3 isoforms, and 14-3-3ζ mediates Tau aggregation in human neuroblastoma M17 cells [31]. 14-3-3ζ has been identified as an oncogene of several tumors, and overexpression of 14-3-3ζ was frequently detected in lung adenocarcinoma tissues, and was significantly associated with lymph node metastasis and adverse outcomes [32]. In the study of prostate cancer, the 14-3-3 family of YWHAZ, which is associated with the prognosis of metastatic prostate cancer, can be used as a target for prostate cancer treatment [33]. Related experiments in breast cancer found that phosphorylation of BAD at S118 stimulates the survival pathway, which in turn phosphorylates BAD at S99, resulting in binding to the 14-3-3 protein, thereby affecting the proliferation of breast cancer tumor cells [34].

CFP1 is closely related to MBD1, MBD2 on the 18q21 chromosome, a region that is often damaged in cancer. CFP1 has a CXXC domain, a highly conserved domain among several proteins, including DNA methyltransferase 1 (DNMT1). It can participate in the regulation of the chromosome 18q21 gene region, but rarely occurs in primary colon cancer and lung cancer [35]. It can be seen through experiments that the DNMT1 CXXC domain can functionally replace the MLL CXXC domain to enable the MLL-AF9 fusion to cause leukemia [36].

### CFP1 may cross-react with 14-3-3 via NF-KB

Through the literature, we can find that 14-3-3 can recruit the gene promoter of the NF-KB pathway, and NF-KB is specifically enriched for histone 3 lysine 4 trimethylation (H3K4me3) by CFP1 and promoter [37]. Our current study suggests that CFP1 and 14-3-3 have opposite effects on prognosis in gastric cancer, both of which are related to the NF-KB pathway. Therefore, we can speculate that the two may affect the cell cycle, cell migration, cell–cell communication and programmed cell death through the NF-KB pathway, which has a certain impact on tumor occurrence and prognosis survival time. Based on this conjecture, we can confirm the relationship between the two through further experiments to judge the prognosis of the patient and possibly guide its treatment. However, the specific results need to be confirmed by further experiments.

## Conclusion

In this study, we analyzed the immunohistochemistry of patient lesions and the effects of two genes in a public database. Immunohistochemistry showed that CFP1 was expressed in the nucleus, while 14-3-3 was mainly present in the cytosol. At the same time, through public database analysis, it can be seen that the effect of CFP1 and 14-3-3 on the survival time of patients with gastric cancer is opposite, that is, patients with high expression of CFP1 have shorter survival time than patients with low expression, while the survival time of gastric cancer patients with low expression of 14-3-3 is shorter than the patients with high expression. This suggests that CFP1 and 14-3-3 have a certain role in gastric cancer and may be a target for treatment.

This study has some limitations. First of all, our sample size is a total of 84, the number is not enough, and a considerable part of the sample was lost during the follow-up process, and the follow-up was not completed. Second, although we borrowed public databases, there are still some shortcomings. Progression-free survival refers to the period of time between the onset of treatment, the onset of disease progression, or the death of any cause. However, because of insufficient clinical data, some patients lost follow-up and we were unable to analyze progression-free survival. The analysis of progression-free survival time is also not possible through public databases, and the combined effects of CFP1 and 14-3-3 are still not clear for the time being, and further experiments are needed to discover and confirm the link between the two. These limitations may have a certain impact on the results of this study, and under the same conditions these restrictions may be overcome if the sample size is large enough.

## Data Availability

Not applicable.

## Abbreviations

GC: gastric cancer
CFP1: CXXC zinc finger protein 1
H3K4me3: histone 3 lysine 4 trimethylation
SET1/SETD1: SET domain containing 1
YWHAZ: tyrosine 3-monooxygenase/tryptophan 5-monooxygenase activation protein zeta
MBD1: methyl-CpG binding domain protein 1
MBD2: methyl-CpG binding domain protein 2
DNMT1: DNA methyltransferase 1
